# Enhancement of insect susceptibility and larvicidal efficacy of Cry4Ba toxin by calcofluor

**DOI:** 10.1186/s13071-018-3110-3

**Published:** 2018-09-20

**Authors:** Somphob Leetachewa, Narumol Khomkhum, Somsri Sakdee, Ping Wang, Saengduen Moonsom

**Affiliations:** 1 Bacterial Protein Toxin Research Cluster, Institute of Molecular Biosciences, Mahidol University, Nakorn-Pathom, 73170 Thailand; 2 Department of Protozoology, Faculty of Tropical Medicine, Mahidol University, Ratchadewee, Bangkok, 10400 Thailand; 3 Department of Entomology, New York State Agricultural Experiment Station, Cornell University, Geneva, NY 14456 USA

**Keywords:** *Bacillus thuringiensis*, Calcofluor, Peritrophic membrane, Permeability, Insect susceptibility

## Abstract

**Background:**

Mosquitoes transmit many vector-borne infectious diseases including malaria, dengue, chikungunya, yellow fever, filariasis, and Japanese encephalitis. The insecticidal δ-endotoxins Cry4, Cry11, and Cyt produced from *Bacillus thuringiensis* have been used for bio-control of mosquito larvae. Cry δ-endotoxins are synthesised as inactive protoxins in the form of crystalline inclusions in which they are processed to active toxins in larval midgut lumen. Previously, we demonstrated that the activated Cry4Ba toxin has to alter the permeability of the peritrophic membrane (PM), allowing toxin passage across PM to reach specific receptors on microvilli of larval midgut epithelial cells, where the toxin undergoes conformational changes, followed by membrane insertion and pore formation, resulting in larval death. A peritrophic membrane (PM)-binding calcofluor has been proposed to inhibit chitin formation and enhance baculovirus infection of lepidopteran *Trichoplusia ni.*

**Methods:**

In this study, *Aedes aegypti* larvae were fed with the calcofluor and Cry4Ba toxin to investigate the effect of this agent on the toxicity of the Cry4Ba toxin.

**Results:**

Calcofluor displayed an enhancing effect when co-fed with the Cry4Ba wild-type toxin. The agent could restore the killing activity of the partially active Cry4Ba mutant E417A/Y455A toward *Ae. aegypti* larvae. PM destruction was observed after larval challenge with calcofluor together with the toxin. Interestingly, calcofluor increased Cry4Ba toxin susceptibility toward semi-susceptible *Culex quinquefasciatus* larvae. However, calcofluor alone or in combination with the toxin showed no mortality effect on non-susceptible fresh-water fleas, *Moina macrocopa*.

**Conclusions:**

Our results suggest that PM may contribute to the resistance of the mosquito larvae to Cry4Ba toxin. The PM-permeability alternating calcofluor might be a promising candidate for enhancing insect susceptibility, which will consequently improve Cry4Ba efficacy in field settings in the future.

## Background

Cry toxins have been used worldwide as bio-insecticide and genetically modified crops because of their environment-friendly properties and killing specificity toward a narrow spectrum of insect larvae. For example, Cry1A and Cry2A are toxic to lepidopterans, Cry3 is noxious to coleopterans, whereas Cry4 and Cry11 are specifically lethal to *Aedes*, *Anopheles* and *Culex* larvae in the family Culicidae [1]. These toxins are produced as parasporal inclusions of 130-kDa protoxins in *Bacillus thuringiensis* (Bt) bacteria. The protoxins are processed by midgut proteases of insect larvae, resulting in active toxins [2, 3]. The active toxins, comprising of three structural domains, pass through the peritrophic membrane (PM), bind to their receptor on the midgut epithelial cell membranes of susceptible insect *via* domain II, oligomerize, and insert domain I into the membrane to create lytic pores, resulting in death of insect larvae by osmotic cell lysis [4–6].

Due to increased planting of Bt transgenic crops around the world [7], insect resistance to Cry toxins has increased, leading to insufficient toxicity of the Cry toxins in the field [8, 9]. Attempts to improve the efficacy of the Cry toxins have been made by toxin combination, genetic modification, and protein engineering [10, 11]. Mutagenesis in the receptor binding domains of Cry1Ab and Cry3A has resulted in significant increase in their toxicities toward lepidopteran and coleopteran insect larvae [12, 13]. Attempts to enhance the toxicity of Cry toxins by focusing on the insect host have been investigated in several insect species. For instance, the introduction of Cry1Ab together with a fragment of the cadherin receptor, CR12-MPED peptide, was found to improve Cry1Ab toxicity toward tobacco hornworm larvae, *Maduca sexta* [14]. Likewise, a 28-kDa APN fragment, AgAPN2tb, showed significant enhancement effect on Cry11Ba toxicity to *Anopheles gambiae* [15].

The PM lining between the midgut epithelial membrane and lumen serves as a protective barrier of the insect larvae from invaders such as bacteria and viruses [16, 17]. Chitin, the major PM component, is secreted by midgut cells to form fibre networks linked by PM proteins secreted from microapocrines [18–20]. Pathogens able to overcome protection of the PM barrier or digest the PM proteins were found to invade the insect hosts successfully. For instance, *Plasmodium gallinaceum* ookinetes produce chitinase enzymes to penetrate the PM to infect midgut cell membrane of *Ae. aegypti* [21]. Combinational treatment of Cry1C toxin and *Serratia marcescens* endochitinase was reported to enhance the activity of the toxin toward *Spodoptera littoralis* larvae [22]. Constitutive expression of chitinase in the Cry1AC producing Bt cells showed an enhancing effect of the Cry1Ac insecticidal activity toward *Sp. exigua* and *Helicoverpa armigera* larvae [23]. The brightening agent, calcofluor, has been utilized for solubilization of proteins from PMs [24]. This chemical has also been shown to inhibit chitin formation and is proposed as an anti-fungal agent [25, 26]. *In vivo*, calcofluor was found to enhance baculovirus infection in *T. ni* [24]. However, there was no synergistic effect of calcofluor on the biological activity of Cry1Ac toward susceptible lepidopteran larvae [27].

We previously reported that PM permeability alteration by Cry4Ba was required for toxin passage across PM of *Ae. aegypti* larvae [28]. Here, we report the enhancing effect of PM solubilizing agent, calcofluor, on Cry4Ba efficacy toward larvae of the susceptible *Ae. aegypti* and the semi-susceptible *Cx. quinquefasciatus*. Our results demonstrated that calcofluor not only increased PM permeability to Cry4Ba toxin but also altered the gross morphology of the PM. However, no mortality effect was observed when calcofluor was tested against non-susceptible insect, water fleas (*Moina macrocopa*). Thus, PM-permeability alteration capability appears to be a critical factor for Cry4Ba toxicity, and calcofluor could be a potential agent to improve the efficacy of Cry4Ba toxin for bio-control of mosquito larvae, which in turn aids to the prevention of vector-borne diseases in the future.

## Methods

### Cry4Ba larvicidal activity assays

Cry4Ba was expressed as previously described [29]. Toxin inclusions were harvested from *Escherichia coli* expressing Cry4Ba. The inclusions were washed, protein concentrations were determined using Bradford’s assays and confirmed by 12%-gel sodium dodecyl sulfate-polyacrylamide gel electrophoresis (SDS-PAGE). The larvicidal activity was determined by feeding fourth-instar larvae of *Ae. aegypti* or *Cx. quinquefasciatus* with 0.125–200 μg/ml of wild-type or mutant Cry4B-toxin inclusions alone or together with 0.1% calcofluor (Sigma-Aldrich, St. Louis, MO, USA). The assay was performed in a 6-well flat bottom plate containing 25 larvae per well, with 100 larvae/assay. The mortality was recorded after a 24 h incubation period at 25 °C. Larvicidal activity of Cry4Ba was represented as either percent mortality or 50% (LC_50_) and 90% (LC_90_) lethal concentrations. The LC_50_ and LC_90_ were estimated by Probit analysis [30].

To determine the minimum exposure time of calcofluor that caused 90% mortality, the fourth-instar larvae of *Ae. aegypti* were pre-incubated with 0.1% calcofluor for 0.25 h, 0.5 h, 1 h, 1.5 h and 2 h. After pre-incubation, the solution was replaced by the toxin solution at LC_90._ Larval death was recorded after 24 h of toxin incubation. Likewise, the minimal incubation time for LC_90_ toxin after larval pre-treatment with calcofluor was also examined. Larvae were incubated with 0.1% calcofluor for 2 h, and then the solution was replaced with the toxin at LC_90_. Larval mortality was recorded at 3 h, 4 h, 6 h and 12 h after toxin incubation. All of the biological assays were performed in triplicates.

### Toxicity testing of calcofluor and Cry4Ba toxin with *M. macrocopa*

*Moina macrocopa* were reared in the laboratory using the modified batch method as described elsewhere [31]. In brief, 30 neonate *M. macrocopa*, which are less than 24 hours-old, were put in an aquarium tank (20 × 15 × 15 cm) containing 3 l of aged tap water. The water fleas were fed with algae suspension (*Chlorella vulgaris*) of 2 × 10^7^ cells per 1 l of water every 48 h. The culture was carried out at room temperature (25 ± 1 °C) with a 16 h photoperiod and gentle aeration of the oxygenated pool water in climatic chamber conditions.

To determine the toxicity of Cry4Ba, the water fleas were tested with 0.1% calcofluor, LC_90_ of Cry4Ba, or in a combination of 0.1% calcofluor and Cry4Ba at LC_90_ in aged tap water containing algae suspension at the same condition as the culture, and compared to untreated fleas. One hundred fleas were used for each condition in a 100 ml beaker containing 50 ml of the treatment solution. The experiment was performed in triplicate and percent mortality was recorded after 24 h.

### PM permeability and immunohistochemistry assays of toxin fed larvae

The fourth-instar larvae of *Ae. aegypti* were fed with 2000 kDa FITC-dextran particles alone or together with wild-type or mutant Cry4Ba toxins at their LC_90_ for 1 h. The PM-permeability alteration by the toxin was measured and indicated by the appearance of 2000 kDa FITC-dextrans, the PM impermeable particles, outside the gut lumen of mosquito larvae, and observed under the fluorescent microscope [28, 32]. To observe the effect of calcofluor on PM permeability, the experiment was performed in the presence of 0.1% calcofluor. Results were analyzed using Student’s t-test and *P*-values less than 0.05 were considered significant.

To determine the binding ability of the mutant toxin, larvae were fed with wild-type or mutant Cry4Ba at LC_90_ for 1 h. Immunohistochemistry assay was performed using HRP-labeled anti-Cry4Ba monoclonal antibody. Colourimetric detection observed signal with 3,3′-diaminobenzidine (DAB) substrate, following manufacturer’s instructions (Sigma Aldrich). The pictures were recorded using a light microscope.

### Hematoxylin and eosin staining of the *Ae. aegypti* larval gut

Larvae were pre-treated with 0.1% calcofluor for 2 h followed by incubation with LC_90_ of the toxin for 1 h. Head and eighth segment were removed from larvae. The dissected larvae were fixed in phosphate-buffered saline (PBS, pH 7.4) containing 4% paraformaldehyde and 4% sucrose for 1 h, and then placed in 4% paraformaldehyde solution containing 10% sucrose overnight at 4 °C. Next, larvae were incubated in a series of ethanol at room temperature for 20 min each; 30%, 50%, 70%, 95%, and twice in absolute ethanol. The section was placed twice in xylene solution for 30 min/each and incubated twice in xylene-paraffin (1:1 v/v) for 1 h/each, before being changed to 100% paraffin (2 × 1 h). The paraffinized section was horizontally sliced, embedded onto a glass slide, and incubated at 37 °C overnight. The tissue slide was rehydrated in PBS for 30 min, before staining with hematoxylin and eosin. The tissue section was washed with PBS 3 times (30 min each). The whole gut and the peritrophic membrane were observed and imaged using a light microscope (Olympus, Tokyo, Japan) or fluorescent microscopes (Carl Zeiss, Jena, Germany). Larvae fed with toxin alone and of the untreated larvae were used as positive and negative controls, respectively.

### Determination of larval PM structure using scanning electron microscopy (SEM)

*Aedes aegypti* larvae were fed for 1 h with 0.1% calcofluor alone, LC_90_ of Cry4Ba inclusions, and the combination of toxin and calcofluor. Fifty of the dissected PMs were washed in distilled water. The PMs were fixed in 2.5% glutaraldehyde in 0.1 M phosphate buffer (pH 7.4) containing 5% glucose (fixing solution) at 4 °C for 2 h. After three washes (10 min each) in fixing solution, the PM samples were dehydrated in a series of ethanol concentrations and finally dried by a critical point drying using Hitachi HCP-2 dryer (Hitachi Koki, Ibaraki, Japan). The dried samples were mounted onto the aluminium stubs, coated with gold particles using an ion coater (K 500, Emitech Ltd., England), and observed at 10 kV on the electron microscopy (JSM-6610LV, JELO Ltd., Japan).

## Results

### Calcofluor reduced the lethal concentrations and exposure time of wild-type Cry4Ba toxin

**Fig. 1:**
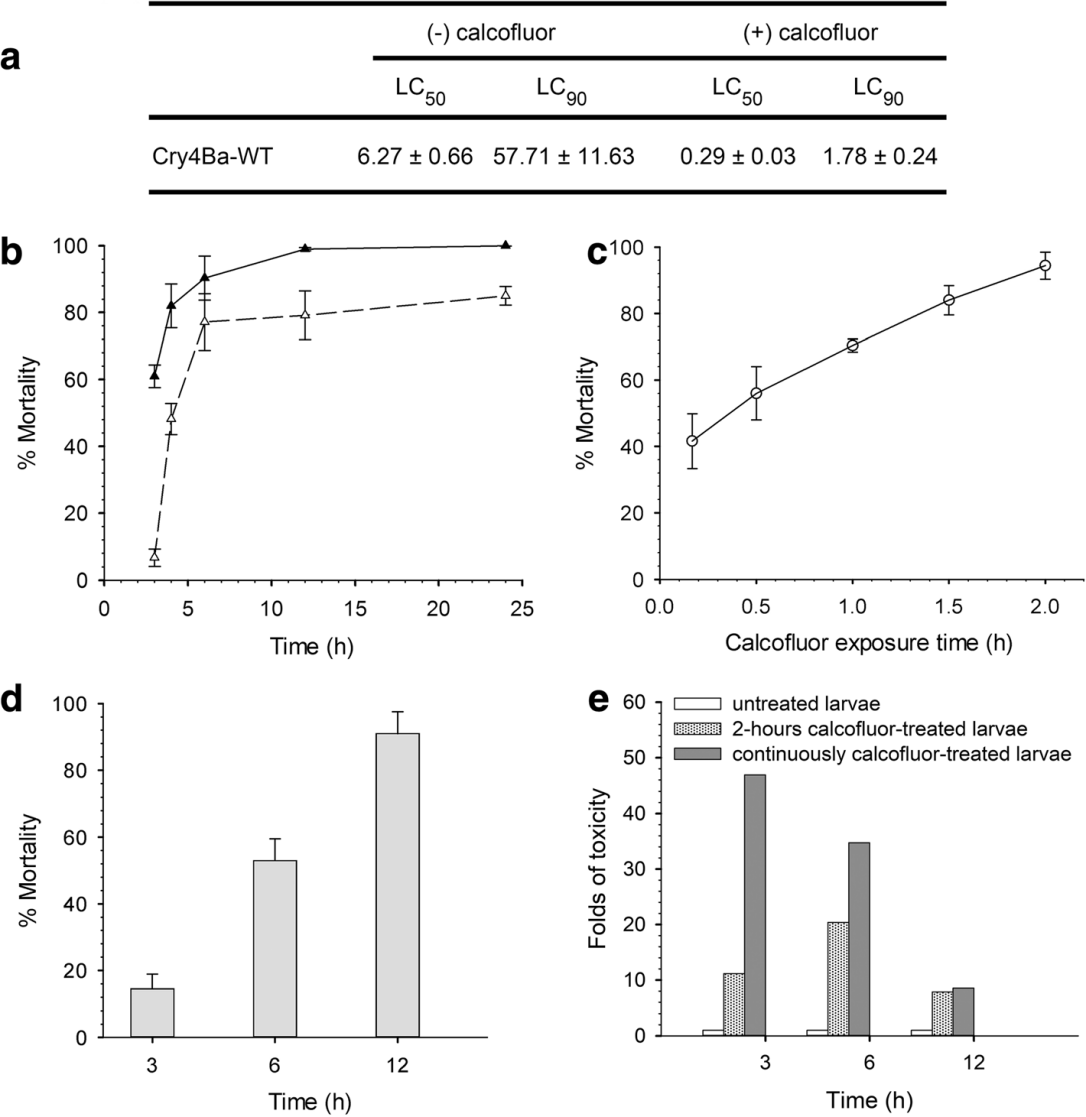
Enhancing effect of calcofluor on the toxicity of wild-type Cry4Ba toward *Ae. aegypti* larvae. Larvae were fed with various concentrations of wild-type Cry4Ba toxin in the presence (+) or absence (-) of calcofluor for 24 h, LC_50_ and LC_90_ were estimated (**a**). The larvae were fed with toxin at LC_90_ alone (dash line), and toxin with calcofluor (solid line) and percent mortality was recorded after 3 h, 4 h, 6 h, 12 h and 24 h incubation period (**b**). Larvae were pre-incubated with 0.1% calcofluor for 0.25 h, 0.5 h, 1 h, 1.5 h and 2 h before 24 h feeding with toxin at LC_90_, and percent mortality was then recorded (**c**). Larvae were pre-exposed with 0.1% calcofluor for 2 h, and then the solution was replaced with toxin inclusion at LC_90_. Percent mortality was recorded after 3 h, 6 h and 12 h and presented as percent mortality + SEM (as T-bars) (**d**). Comparison of toxicity of Cry4Ba toxin toward larvae, which were 2 h pre-exposed with 0.1% calcofluor and continuously fed with the calcofluor together with LC_90_ of the toxin for 3 h, 6 h, and 12 h, and represented as folds of toxicity compared to calcofluor untreated larvae (**e**). *Abbreviations*: SEM, standard error of the mean

The effect of calcofluor on Cry4Ba toxicity was determined by feeding the fourth-instar larvae of *Ae. aegypti* with Cry4Ba-toxin inclusions alone or in combination with calcofluor for 24 h. Compared to those without calcofluor, the co-feeding of wild-type toxin with calcofluor reduced toxin concentrations needed for LC_50_ by 6 folds (from 6.27 ± 0.66 μg/ml to 0.29 ± 0.03 μg/ml) and concentration required for LC_90_ by 50 folds (from 57.71 ± 11.63 μg/ml to 1.78 ± 0.24 μg/ml), respectively. The effect of calcofluor was further determined as the function of time by feeding the larvae with calcofluor and Cry4Ba-toxin inclusions at *c.*2 μg/ml or toxin alone at ca. 60 μg/ml according to the LC_90_ of calcofluor-treated and untreated conditions in Fig. 1a. Larvae fed with toxin alone for 3 h, 4 h, 6 h and 12 h demonstrated mortality as mean ± SD of 6.7 ± 2.5%, 48.2 ± 4.6%, 77.2 ± 8.5% and 79.2 ± 7.3%, and nearly 90% after 24 h, respectively (Fig. 1b, dashed line). By co-feeding with 0.1% calcofluor, larval mortality was 61.0 ± 8.4% within 3 h, increased sharply to 82.0 ± 6.6% and 90.3 ± 6.6% after 4 h and 6 h, respectively, and gradually rose to 99.5 ± 0.5% within 12 h (Fig. 1b, solid line). Larvae were pre-challenged with calcofluor at various time points before the toxin feeding at LC_90_. Larvae pre-challenged with calcofluor for 0.25 h, 0.5 h, 1 h, 1.5 h and 2 h, showed mortality of 41.6 ± 8.2%, 56.0 ± 8.0%, 70.4 ± 2.0%, 84.0 ± 4.4% and 94.4 ± 4.1% after 24 h of toxin feeding, respectively (Fig. 1c). Larvae pre-challenged with calcofluor for 2 h and then fed with LC_90_ of Cry4Ba for 3 h, 6 h and 12 h showed mortality of 16.0 ± 3.0%, 54.0 ± 6.6% and 90.0 ± 6.6%, respectively (Fig. 1d). Enhancement of Cry4Ba toxicity by calcofluor was investigated further in untreated larvae in comparison to those temporarily exposed with the calcofluor for 2 h before toxin feeding, and continuously treated with calcofluor and toxin (Fig. 1e). After 3 h of toxin treatment, the continuously calcofluor-treated and the 2 h calcofluor-exposed larvae exhibited 46- and 12-fold higher mortality than the calcofluor untreated group, respectively. At 6 h of toxin incubation, mortality of the 2 h calcofluor exposed larvae was 21-fold higher than the calcofluor untreated larvae. However, this mortality was 14-fold lower than the larvae that were continuously fed with the combination of calcofluor and toxin. At 12 h of toxin treatment, mortality of 2 h calcofluor exposed larvae were close to those continuously fed with calcofluor and toxin, but 8- and 9-fold higher than non-treated larvae, respectively.

### Calcofluor restored the toxicity of the partially active E417A/Y455A (EY)-mutant Cry4Ba toxin

**Fig. 2:**
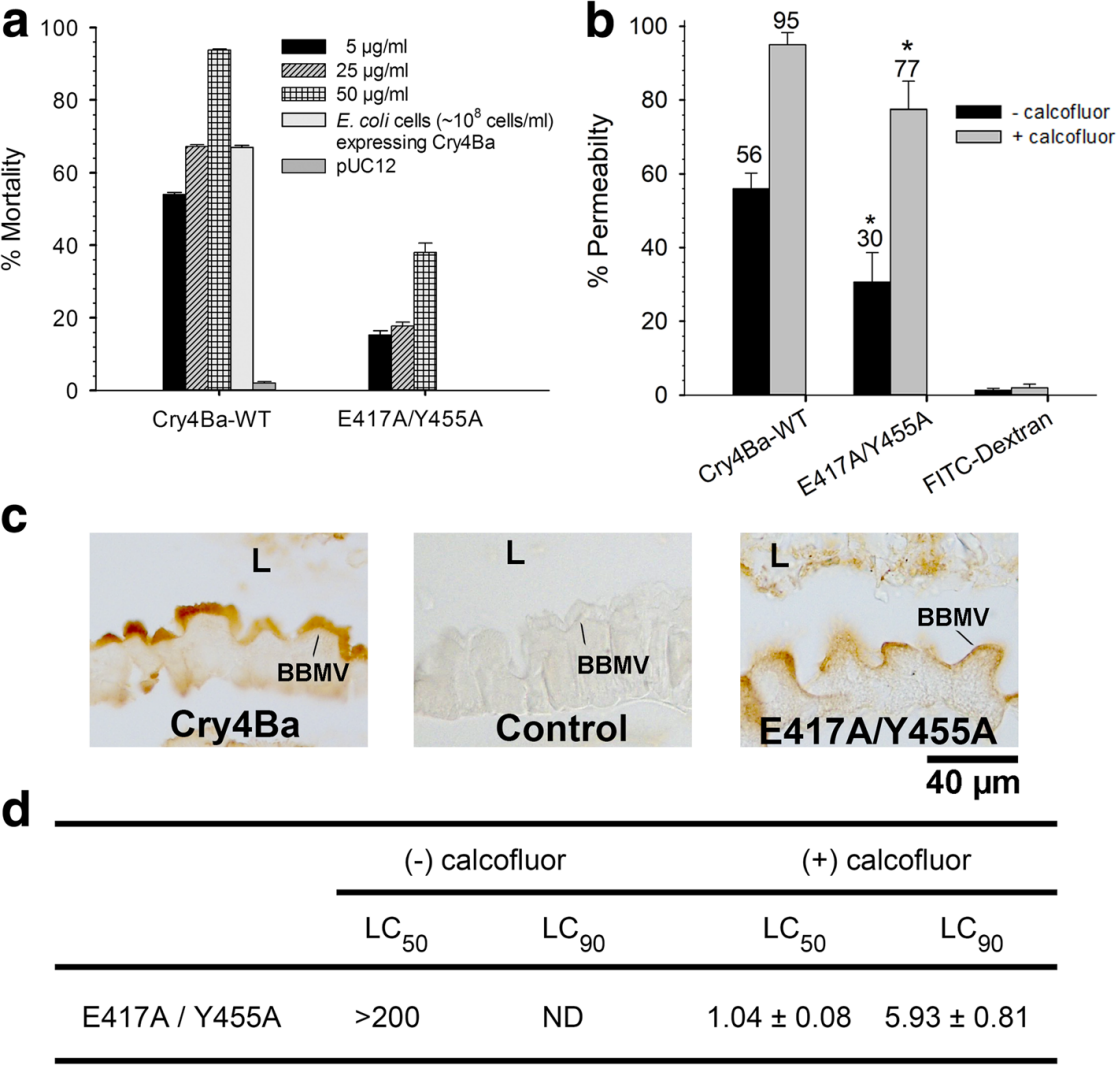
Toxicity and biological activities of EY mutant toxin in the presence and absence of calcofluor. Larvae were fed with toxin inclusion bodies of wild-type Cry4Ba and EY mutant toxin at 5, 25 and 50 μg/ml, *E. coli* cells expressing Cry4Ba, and *E. coli* cells harbouring expressing plasmid pUC12. Percent mortality was represented as the mean + SEM (**a**). PM perturbation ability of Cry4Ba toxin was evaluated by feeding the larvae with PM impermeable 2000 kDa dextran (conjugated with FITC) alone or in combination with wild-type Cry4Ba and EY mutant toxin at their LC_90_ concentrations. Percent PM permeability was represented as % of larvae with fluorescent signal outside the gut lumen after one h treatment (**b**). The tissue sections of treated larvae were immuno-stained with mouse anti-Cry4Ba monoclonal antibody and HRP-linked anti-mouse immunoglobulins (**c**). LC_50_ and LC_90_ of EY mutant toxin were determined after 24 h treatment in the presence or absence of calcofluor and presented as μg/ml ± SEM (**d**). The lines point out the BBMV that are positively stained with wild-type Cry4Ba and EY mutant toxins. Control is untreated larval gut tissue. *Abbreviations*: L, lumen; BBMV, brush border microvilli

In the absence of calcofluor, *Ae. aegypti* larvae exhibited mortality of about 47%, 74%, 97% and 74% upon feeding with wild-type Cry4Ba at concentrations 5, 25, and 50 μg/ml, and 10^8^ of toxin-expressing *E. coli* cells, respectively (Fig. 2a). EY mutant toxin showed very low toxicity to the larvae compared to wild-type Cry4Ba (Fig. 2a). The mutant toxin also exhibited approximately 50% in perturbation ability of PM permeability compared to wild-type Cry4Ba (Fig. 2b). However, it exhibited the similar binding ability to the brush border membrane of the larval midgut as the wild-type Cry4Ba (Fig. 2c). When co-fed with calcofluor, PM perturbation ability of the EY mutant and wild-type Cry4Ba rose to 95% and 77%, respectively (Fig. 2b). Furthermore, the LC_50_ and LC_90_ of the EY mutant toxin were significantly decreased from > 200 μg/ml to 1.04 ± 0.08 μg/ml (t-test: t_(4)_ = 9.127, *P* = 0.0004) and 5.93 ± 0.81 μg/ml, respectively (Fig. 2d).

### Calcofluor induced morphological changes of PM

**Fig. 3:**
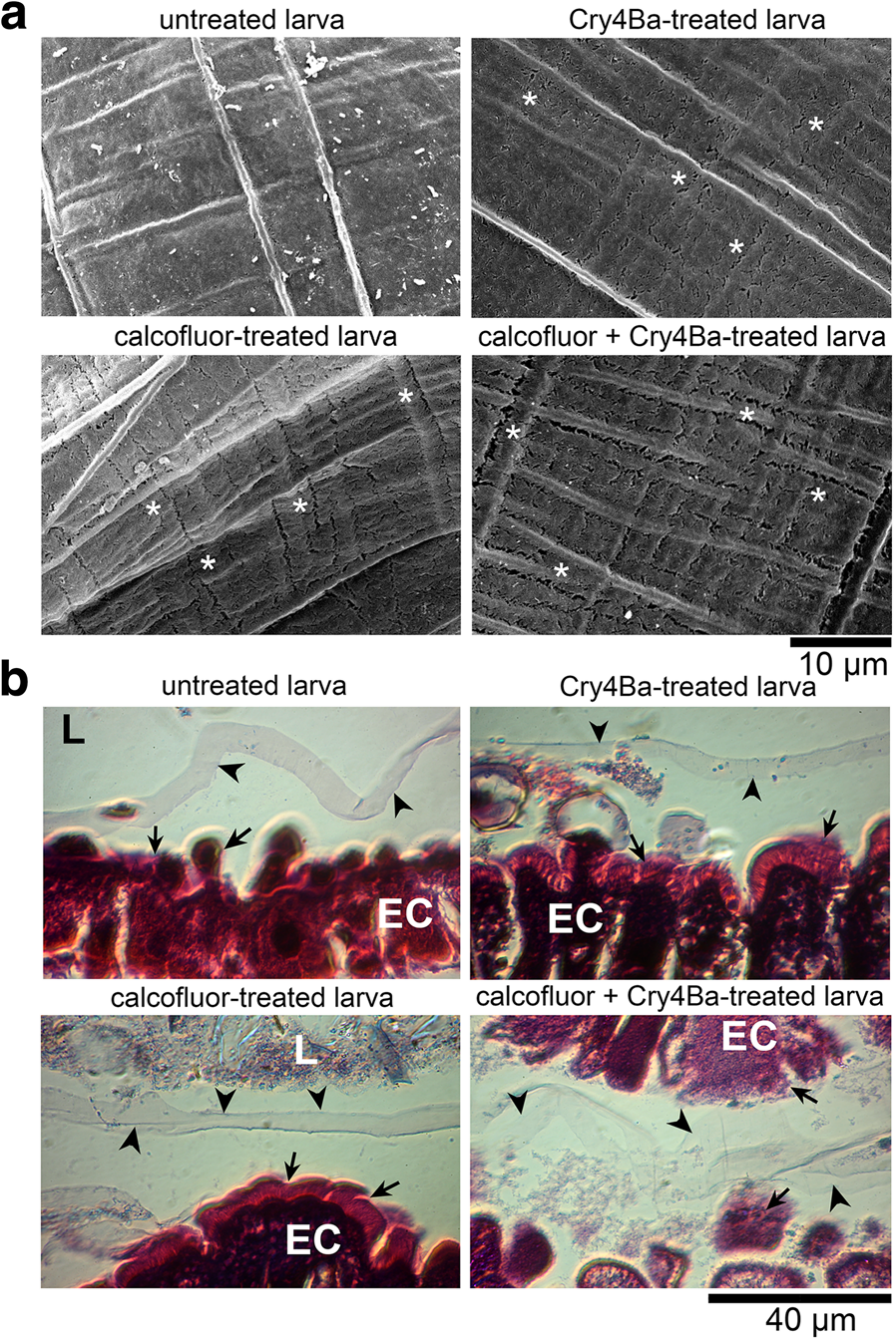
PM topographies of calcofluor and non-calcofluor treated *Ae. aegypti* larvae. PMs isolated from untreated larvae, calcofluor-treated larvae, Cry4Ba-treated larvae, and toxin and calcofluor co-fed larvae were analyzed with SEM (**a**). Tissue sections of treated larvae were stained with H&E and examined under a light microscope (**b**). Asterisks mark cracks along thick lines of PMs. Arrows indicate microvilli of the midgut epithelial cells and arrowheads present PM after staining with H&E. *Abbreviations*: L, lumen; EC, epithelial cells of the midgut

The scanning electron microscopy (SEM) demonstrated smooth surface of the PM with well-organised micro-fibril observed as thick lines in untreated larva (Fig. 3a). Slight cracks were observed in the PM of larvae treated with calcofluor or with Cry4Ba alone. These cracks were visible along a thick-line component of the PM in larvae treated with toxin together with calcofluor (Fig. 3a). The H&E stained gut tissues showed that gross morphology of the midgut-brush border microvilli (BBMV) of calcofluor-treated larvae remained intact as of untreated larvae (Fig. 3b), whereas BBMVs of the larvae treated with toxin alone, and toxin together with calcofluor presented mild (epithelial cells and BBBV were swollen and basolateral cell junctions were separated) to severe (epithelial cells and BBMV were disrupted entirely) damage of the BBMVs and midgut cells, respectively (Fig. 3b). However, gross morphology of the PMs was not different among H&E stained tissue samples.

### Calcofluor significantly improved susceptibility of *Cx. quinquefasciatus* larvae to the Cry4Ba toxin

**Fig. 4:**
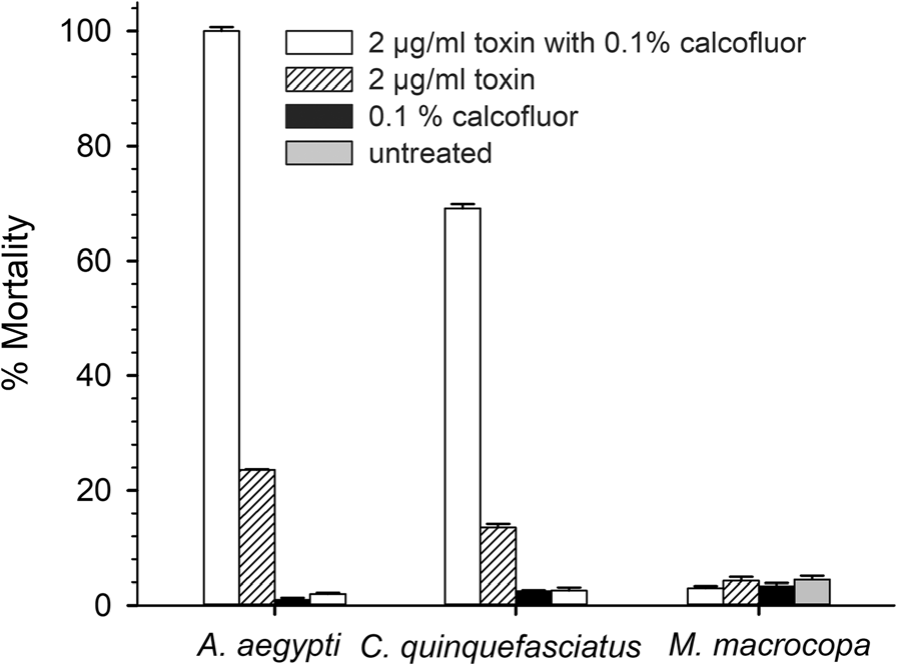
Cry4Ba activity toward *Ae. aegypti*, *Cx. quinquefasciatus* and *M. macrocopa* in the presence and absence of calcofluor. The larvae were fed with 0.1% calcofluor, Cry4Ba toxin at LC_90_ and the toxin in combination with calcofluor. Percent mortality of mosquito larvae was observed after 24 h and compared to untreated larvae. Results are shown as percent mortality + SEM (T- bars) from triplicate experiments

Along with *Ae. aegypti* larvae, the fourth-instar larvae of *Cx. quinquefasciatus* and the non-susceptible water flea *M. macrocopa* were fed with Cry4Ba toxin at LC_90_, calcofluor and LC_90_ of the Cry4Ba toxin together with calcofluor for 24 h. The percent mortality of *Cx. quinquefasciatus* larvae were 2.5 ± 0.14% and 13.5 ± 0.62% when fed 0.1% calcofluor and with toxin alone, respectively (Fig. 4). In the toxin combination with calcofluor, there was a sharp increase in mortality of *Cx. quinquefasciatus* larvae to 69.1 ± 0.78%. However, there was no mortality effect of calcofluor and the calcofluor-Cry4Ba toxin combination to non-susceptible water fleas (Fig. 4).

## Discussion

The insect PM is comprised of proteins, proteoglycans, and a chitin network that serves as a protective barrier for physical damage by food particles, as well as compartmentalization and permeability of insect-gut digestive enzymes and other solutes [20]. When fed to *Ae. aegypti* larvae, Cry4Ba gradually expressed its toxicity in a sigmoidal shape three hours after feeding and plateaued after six. Previously, Cry1Ac and Cry4Ba were reported to be associated with larval PM [27, 28]. This Cry4Ba-PM association might trigger secondary host response or signaling transduction as proposed by the toxicity mechanism of lepidopteran-specific Cry toxins [33, 34], leading to delayed toxicity. Cry4Ba toxin was previously shown to alter PM permeability to cross over PM and bind to the midgut membrane for its toxicity toward *Ae. aegypti* larvae [28]. Alternatively, the delayed toxicity might be due to the protective threshold of the larval PM for Cry4Ba toxin. Destruction of PM structure and components by chitinase [16] and bel protein produced from Bt [17] were found to promote toxicity of Cry1A protein toward susceptible insect larvae. In this study, when calcofluor was administered, the toxicity of Cry4Ba was enhanced, as shown by a 50-fold decrease in toxin dosage to achieve its LC_90_. This result is consistent with the positive effect of calcofluor on Cry1Ca toward *Mamestra brassicae* larvae [30] and baculovirus infections in *T. ni* [24, 35, 36]. *Aedes aegypti* larval mortality was reached 60% and 90% three and 12 hours after calcofluor co-treatment with LC_90_ of Cry4Ba, respectively. The result also revealed the binding threshold of calcofluor on PM, which can be overcome in around three hours to set out the toxin enhancing effect. Temporary exposure of larvae with calcofluor, for at least 15 min (0.25 h) before toxin incubation, was also able to enhance Cry4Ba toxicity. However, Cry4Ba toxin continuously co-fed with calcofluor exhibited significantly higher toxicity than the brief exposure, suggesting that calcofluor might bind to larval PM in a semi-irreversible manner. In previous reports, PMs of lepidopteran larvae were disrupted and fragmented upon treatment with calcofluor, and completely recovered after removal of the chemical [24, 27]. Although the amount of calcofluor and toxin exposure time applied for *Ae. aegypti* larvae in the current study were much lower than in previous reports with lepidopteran larvae, the enhancing effect of calcofluor on Cry4Ba toxicity remained observable. Therefore, this study reveals the thermodynamic process of calcofluor action in dipteran larvae (semi-irreversible), which differs from lepidopteran larvae (reversible). Beside the enhancing effect, a combination of calcofluor with Cry1Ac or Cry1Ca was found to inhibit their toxicities toward *M. sexta* larvae [27]. It is important to note that PM is composed of proteins and glycoproteins embedded in the chitin network [37, 38]. However, the proportion of protein and chitin components varies in different insect species [20, 39, 40]. Therefore, the difference in PM structure and components, as well as the toxic mechanism of the Cry toxins, possibly result in different degree of calcofluor effect on the toxicity of Cry toxins toward insect larvae.

Amino acids at positions Glu417(E) and Tyr455 (Y) of Cry4Ba domain II were proposed to be involved in toxin binding to BBMV of *Ae. aegypti* larval midgut [41], while residue Arg158 of domain I was shown to be responsible for toxin insertion into PM and midgut membrane [28]. Compared to the wild-type Cry4Ba, the mutant EY was defective in PM permeability alteration, resulting in near loss of toxicity toward *Ae. aegypti* larvae. When administered with calcofluor, the mutant EY was able to recover its toxicity, and all of the treated larvae were permeable to the supposedly non-permeable 2000 kDa dextran particles. Thus, current results revealed the additional function of amino acids Glu417 and Tyr455 of the Cry4Ba toxin in permeability alteration of *Ae. aegypti* larval PM.

We investigated further whether the increased toxicity was due to changes in PM's gross morphology by calcofluor. It was found that calcofluor alone caused the degeneration of PM network than the epithelial membrane of the larval midgut. This is consistent with the finding in lepidopteran larvae, where PM was not intact after calcofluor incubation [27]. Fully formed PM of *Ae. aegypti* larvae were assembled by electron-dense knots and streaks connected by one to two electron-lucent layers formed in honeycomb-like holes [42]. The electron-dense layers (thick lines in SEM) are at least composed of proteoglycans [42], while the electron-lucent layers (thin lines in SEM) are believed to contain chitin microfibrils [43]. Electron micrographs of calcofluor-treated larvae demonstrated the destruction of the PM at areas of thin lines. Severe damage was observed in PMs of larvae treated with calcofluor in combination with Cry4Ba toxin. It was first presented here that calcofluor was able to destroy chitin fibrils of mosquito larval PM. Due to binding specificity of calcofluor to cellulose and chitin components of insect PM [38], its binding might promote accessibility of chitin fibril for digestion by gut residing chitinases or deposition of the peritrophins and other chitin-binding proteins. These events might lead to the increase in PM permeability to Cry4Ba [28], while performing capability of Cry4Ba, and cause severe damage to PM and midgut epithelium, contributing to the death of calcofluor-toxin-treated larvae.

A hallmark of Cry toxins is their killing specificity toward a narrow spectrum of insect hosts. Cry4Ba toxin is highly toxic to *Aedes* and *Anopheles* [44] and partially harmful to *Culex* mosquito larvae. Previous substitution of amino acid Asp 454 on β10- β11 loops of Cry4Ba domain II with Lys or Arg (D454K and D454R) was found to increase Cry4Ba toxicity toward *Cx. quinquefasciatus* larvae [45]. In this study, treatment of *Cx. quinquefasciatus* larvae with wild-type Cry4Ba with calcofluor significantly improved larval susceptibility to the toxin and expressed higher mortality than those treated with toxin alone. However, the toxicity of Cry4Ba toward *Cx. quinquefasciatus* larvae were relatively low compared to that of *Ae. aegypti* larvae. Peters [46] found that electron-dense layers at the lumen side of *Cx. pipiens* larval PM is discontinuously interrupted with fine pores, whereas those of *Ae. aegypti* are continuous with a granular appearance at the midgut membrane side. Although the chitin fibrils in electron-lucent layers of *Cx. pipiens* larval PM is arranged in an orthogonal texture similar to *Ae. aegypti*, those portions are much larger than the PM of *Ae. aegypti* [46]. These differences in PM ultra-structures may attribute to the degrees of larval susceptibility to Cry4Ba toxin between these two closely related mosquito species. Despite the enhancing effect of calcofluor on Cry4Ba, it showed no mortality toward water fleas (*M. macrocopa*). This result demonstrated that calcofluor is safe for a zooplankton *M. macrocopa*, which is known as an environmental parameter as the primary level of the food chain in the ecosystem [47–49]. Also, calcofluor has relatively low toxicity to fish, mammals and humans and has not been found to be carcinogenic or mutagenic to humans [50].

## Conclusions

Calcofluor can function as an enhancing agent for Cry4Ba toxicity by increasing the susceptibility of *Ae. aegypti* and *Cx. quinquefasciatus* larvae, resulting in a reduction of both toxin concentration and exposure time. This chemical exhibited no toxicity toward non-susceptible water fleas. Thus, this study suggested the possible field application of Cry4Ba-toxicity enhancing calcofluor to improve the efficacy of bio-control of mosquito vectors soon.

## Abbreviations

BBMV: Brush border microvilli
Bt: *Bacillus thuringiensis*
CR12-MPED: 12 cadherin repeats-membrane proximal extracellular domain
DAB: 3,3′-diaminobenzidine
FITC: Fluorescein isothiocyanate
H&E: Hematoxylin and eosin
LC_50_: Lethal concentration required to kill 50% of the population
LC_90_: Lethal concentration required to kill 90% of the population
PBS: Phosphate-buffered saline
PM: Peritrophic membrane
SDS-PAGE: Sodium dodecyl sulfate-polyacrylamide gel electrophoresis
SEM: Scanning electron microscope
